# DNA Thermo-Protection Facilitates Whole-Genome Sequencing of Mycobacteria Direct from Clinical Samples

**DOI:** 10.1128/JCM.00670-20

**Published:** 2020-09-22

**Authors:** Sophie George, Yifei Xu, Gillian Rodger, Marcus Morgan, Nicholas D. Sanderson, Sarah J. Hoosdally, Samantha Thulborn, Esther Robinson, Priti Rathod, A. Sarah Walker, Timothy E. A. Peto, Derrick W. Crook, Kate E. Dingle

**Affiliations:** a Nuffield Department of Clinical Medicine, John Radcliffe Hospital, Oxford University, Oxford, United Kingdom; b National Institute for Health Research (NIHR) Oxford Biomedical Research Centre, John Radcliffe Hospital, Oxford, United Kingdom; c Microbiology Department, Oxford University Hospitals NHS Trust, Oxford, United Kingdom; d Respiratory Medicine Unit, Nuffield Department of Medicine, John Radcliffe Hospital, University of Oxford, Oxford, United Kingdom; e PHE National Mycobacteria Reference Service—North and Central, Birmingham Public Health Laboratory, Birmingham, United Kingdom; Carter BloodCare and Baylor University Medical Center

**Keywords:** DNA sequencing, mycobacteria, *Mycobacterium tuberculosis*, nanopore DNA sequencing, clinical diagnostics, direct-from-sample sequencing

## Abstract

Mycobacterium tuberculosis is the leading cause of death from bacterial infection. Improved rapid diagnosis and antimicrobial resistance determination, such as by whole-genome sequencing, are required. Our aim was to develop a simple, low-cost method of preparing DNA for sequencing direct from M. tuberculosis-positive clinical samples (without culture). Simultaneous sputum liquefaction, bacteria heat inactivation (99°C/30 min), and enrichment for mycobacteria DNA were achieved using an equal volume of thermo-protection buffer (4 M KCl, 0.05 M HEPES buffer, pH 7.5, 0.1% dithiothreitol [DTT]). The buffer emulated intracellular conditions found in hyperthermophiles, thus protecting DNA from rapid thermodegradation, which renders it a poor template for sequencing. Initial validation experiments employed mycobacteria DNA, either extracted or intracellular. Next, mock clinical samples (infection-negative human sputum spiked with 0 to 10^5^ Mycobacterium bovis BCG cells/ml) underwent liquefaction in thermo-protection buffer and heat inactivation. DNA was extracted and sequenced. Human DNA degraded faster than mycobacteria DNA, resulting in target enrichment. Four replicate experiments achieved M. tuberculosis detection at 10^1^ BCG cells/ml, with 31 to 59 M. tuberculosis complex reads. Maximal genome coverage (>97% at 5× depth) occurred at 10^4^ BCG cells/ml; >91% coverage (1× depth) occurred at 10^3^ BCG cells/ml. Final validation employed M. tuberculosis-positive clinical samples (*n* = 20), revealing that initial sample volumes of ≥1 ml typically yielded higher mean depths of M. tuberculosis genome coverage, with an overall range of 0.55 to 81.02. A mean depth of 3 gave >96% 1-fold tuberculosis (TB) genome coverage (in 15/20 clinical samples). A mean depth of 15 achieved >99% 5-fold genome coverage (in 9/20 clinical samples). In summary, direct-from-sample sequencing of M. tuberculosis genomes was facilitated by a low-cost thermo-protection buffer.

## Abstract (precis)

Mycobacterium tuberculosis is the leading cause of death from bacterial infection. Improved rapid diagnosis and antimicrobial resistance determination, such as by whole-genome sequencing, are required. Our aim was to develop a simple, low-cost method of preparing DNA for sequencing direct from M. tuberculosis-positive clinical samples (without culture). Simultaneous sputum liquefaction, bacteria heat inactivation (99°C/30 min), and enrichment for mycobacteria DNA were achieved using an equal volume of thermo-protection buffer (4 M KCl, 0.

## INTRODUCTION

Mycobacterium tuberculosis is the leading bacterial cause of death from infection, with the World Health Organization (WHO) estimating that 10 million new tuberculosis (TB) cases and 1.2 million deaths occurred worldwide in 2018 (1). In addition, 5 to 10% of an estimated 1.7 billion people with latent TB infections are at risk of progressing to active disease. The greatest TB burden occurs in under-resourced regions of Southeast Asia, Africa, and the Western Pacific. The number of reported cases at 7 million (1) is considerably less than the estimated total. Consequently, the development of diagnostic methods to identify “missing” cases is a global priority (2, 3). Rapid diagnosis and antimicrobial resistance determination are essential to ensure appropriate TB treatment and control, particularly in light of increasing drug resistance (4, 5).

The application of DNA sequencing to mycobacteria molecular diagnostics yields clinically and epidemiologically valuable information. The utility increases with the proportion of genome obtained, from detection to speciation and antimicrobial resistance prediction to phylogenetic and evolutionary insights. This allows whole-genome sequencing (WGS) to outperform other rapid molecular methods (such as GeneXpert MTB/RIF; Cepheid, Solna, Sweden) (6–9).

WGS of M. tuberculosis from early positive cultures offers rapid results compared to those of traditional culture-based methods, which take ≤80 days. The implementation in England of routine mycobacteria sequencing from early positive cultures provides WGS (using the Illumina platform) in 3 to 4 weeks, together with antimicrobial resistance predictions (6, 10). Time to WGS could be reduced if sequencing was performed routinely direct from sample, without culture, and would particularly benefit regions with high TB burdens. A potentially suitable sequencing platform is the Oxford Nanopore Technologies (ONT) device, which is compact, portable, and powered using a laptop USB port. It can be operated in varied and challenging locations (11–14) lacking air-conditioned laboratories or reliable power supplies. However, important sample-preparation issues remain to be solved before direct-from-sample M. tuberculosis sequencing becomes routine using any of the available platforms. First, the essential laboratory health and safety requirement to heat inactivate samples causes extensive DNA degradation (15), and template of sufficient quantity and quality for sequencing is rarely recovered. Second, the proportion of mycobacteria DNA in sputum is as low as 0.01% (16), yielding genome coverage of 0.002 to 0.7× (17), without some form of target DNA enrichment.

Available methods for preparing DNA for direct-from-sample sequencing of M. tuberculosis are complex, costly, and have not been evaluated with the ONT platform. Examples include M. tuberculosis enrichment using SureSelect hybridization and amplification (Agilent, USA), which yielded 90% to complete genome assemblies, allowing antimicrobial susceptibility prediction (18, 19) and informing treatment for a patient in real time (20). An alternative used kit-based depletion of nontarget DNA (17) and obtained wide variation in genome coverage (<12% to >90%). Both approaches included heat inactivation, the former at 80°C for 50 min and the latter at 95°C for 30 min (16, 18), but no steps to mitigate the resultant damage to DNA were taken. Reliance on commercial kits inflates cost, shipping, and storage requirements, making these direct-from-sample approaches less attractive in settings that could benefit most, and these methods have not been widely adopted.

Our aim was to develop a simple, robust, low-cost method of preparing DNA of sufficient quality for sequencing, directly from clinical samples. Heat inactivation was essential, but culture and DNA amplification were excluded. Extracted DNA was to be sequenced using the ONT platform because its robust portability (11–14) is relevant to our target settings and its sequencing of longer input DNA molecules provides an effective test of extracted DNA quality. The protocol was to be immediately transferable to two high burden settings for field testing, India and Madagascar.

## MATERIALS AND METHODS

### Research ethics statement.

The protocol for this study was approved by the London—Queen Square Research Ethics Committee (17/LO/1420). Human samples were collected under approval of East Midlands Research Ethics Committee (08/H0406/189), and all subjects gave written informed consent in accordance with the Declaration of Helsinki.

### Mock clinical samples for method development.

A model system comprising standardized mock clinical samples was established by pooling infection-negative human sputum samples and spiking with enumerated Mycobacterium bovis BCG Pasteur strain at known concentrations.

### (i) Culture and enumeration of BCG cells.

Culture conditions were optimized to yield mostly single BCG cells. Freshly prepared Bactec mycobacterial growth indicator tubes (MGIT) (Becton, Dickinson, Wokingham, UK) were inoculated sparsely with 10 μl BCG Pasteur frozen stock. After 30 days incubation at 37°C, the culture was vortexed vigorously. Larger clusters of BCG cells were allowed to settle for 10 min. Fresh MGIT tubes containing 0.5% Tween 80 (Acros Organics, Geel, Belgium) to encourage nonclustered cell growth (21) were inoculated using 200 μl from the top of the “settled” BCG culture. After incubation at 37°C for 18 days, cells were harvested and counted. Cells from 1 ml were pelleted by centrifugation for 10 min (13,000 rpm), resuspended in 100 μl crystal violet stain (Pro Lab Diagnostics, Birkenhead, UK), and then counted using a Petroff-Hausser counting chamber (Hausser Scientific, Horsham, PA, USA). Enumerated BCG cells were stored at −20°C in 1-ml aliquots until required.

### (ii) Combining enumerated BCG cells with infection-negative sputum.

Human sputum samples were obtained anonymously from asthmatic patients. Up to 10 samples were pooled and then liquefied by treatment with an equal volume of 2× strength thermo-protection buffer (4 M KCl, 0.05 M HEPES buffer, pH 7.5 [Sigma-Aldrich, MO, USA], 0.1% dithiothreitol [DTT] [Roche, UK], nuclease-free molecular biology grade water) to ensure a final concentration of 2 M KCl. Fresh buffer was made weekly and stored in the dark at 4°C. Sputum was incubated at 37°C with occasional vortexing until liquefaction was complete.

Enumerated BCG stock was thawed as needed, and a 10-fold dilution series was made in phosphate-buffered saline (PBS) from 10^5^ to 10^1^ cells per 200 μl. The dilution series was spiked into 800-μl aliquots of the liquefied sputum in 2-ml screw-cap tubes to make 1-ml mock clinical samples. Microscopy was performed on these mock samples using Ziehl-Neelsen (ZN) staining and GeneXpert semiquantitative, cartridge-based PCR (Cepheid, Solna, Sweden) for MTB/RIF Ultra according to the manufacturer’s instructions.

### Validation of mycobacteria heat inactivation.

A validation experiment was performed to confirm that viable mycobacteria did not survive heating at 99°C for 30 min in thermo-protection buffer (Table 1). Control samples prepared in parallel were incubated for 30 min at room temperature. To assess mycobacteria viability postheating, each sample was added to a freshly prepared MGIT tube. These were checked regularly for mycobacteria growth during incubation at 37°C for 8 weeks (or until positive). Lowenstein-Jensen slopes were also inoculated for the heat-treated samples.

**TABLE 1:** Heat inactivation validation

Sample type^*a*^	Mycobacteria cells^*b*^	Heat treatment	Time to positive MGIT culture (days)
Pooled negative human sputum (1 ml) liquefied with thermo-protection buffer containing DTT	M. tuberculosis H37Rv	Room temp, 30 min (control)	10
	M. tuberculosis H37Rv	99°C, 30 min	Negative
	BCG Pasteur	Room temp, 30 min (control)	18
	BCG Pasteur	99°C, 30 min	Negative
Sputasol-treated sputum^*c*^ (1 ml) to which an equal volume of thermo-protection buffer was added	M. tuberculosis H37Rv	Room temp, 30 min (control)	7
	M. tuberculosis H37Rv	99°C, 30 min	Negative
	BCG Pasteur	Room temp, 30 min (control)	8
	BCG Pasteur	99°C, 30 min	Negative
Positive MGIT culture (0.5 ml) plus equal volume thermo-protection buffer	M. tuberculosis H37Rv	Room temp, 30 min (control)	2
	M. tuberculosis H37Rv	99°C, 30 min^*d*^	Negative
	BCG Pasteur	Room temp, 30 min (control)	3
	BCG Pasteur	99°C, 30 min^*d*^	Negative
Positive MGIT culture (1 ml) spun down; pellet resuspended in 1 ml thermo-protection buffer	M. tuberculosis H37Rv	Room temp, 30 min (control)	4
	M. tuberculosis H37Rv	99°C, 30 min	Negative
	BCG Pasteur	Room temp, 30 min (control)	3
	BCG Pasteur	99°C, 30 min	Negative

a The final concentration of KCl used in each heat inactivation experiment was 2 M.

b Spiking inoculum for sputum comprised live cultured BCG or M. tuberculosis H37Rv cells prepared by pelleting cells from 1 ml MGIT culture by centrifugation at 13,000 rpm for 10 min and then resuspending in PBS (1 ml). One drop was used as the inoculum.

c Sputum samples received by the Clinical Microbiology Laboratory, John Radcliffe Hospital, Oxford, without a request for TB testing were decontaminated by treatment with 4% NaOH (E & O Laboratories Ltd, Bonnybridge, Scotland), neutralized, spun down, and resuspended in 1 ml Sputasol. They were then spiked with 1 drop of inoculum.

d A precipitate formed on heating with thermo-protection buffer, possibly comprising salt/antibiotics/medium components.

### Clinical samples.

Mycobacteria-positive clinical respiratory samples comprised sputum samples (*n* = 16), a bronchoalveolar lavage (BAL) specimen (*n* = 1), and lymph node biopsies (*n* = 3). The latter underwent “beating” with large glass beads in saline solution for routine diagnostic testing prior to receipt. Samples were submitted for routine testing at Birmingham Heartlands Hospital NHS Foundation Trust, Birmingham, United Kingdom (*n* = 6), or the Clinical Microbiology Laboratory, John Radcliffe Hospital, Oxford University NHS Foundation Trust, Oxford (*n* = 14). Oxford samples were treated with an equal volume of Sputasol (Oxoid Limited, Basingstoke, UK) prior to receipt and were stored at 4°C. Samples from Birmingham comprised untreated sputum shipped overnight on ice to Oxford, after which they were stored at 4°C. Prior to receipt, microscopy (with auramine staining) had yielded acid-fast bacilli scores of +1 to +3 and/or a positive MTB/RIF Ultra GeneXpert result (Cepheid, Solna, Sweden). Samples were used only after routine diagnostic tests had been completed; therefore, sample quality (available volume, storage time, etc.) varied. Samples were processed as soon as possible after receipt.

### Clinical sample liquefaction and heat inactivation.

All available clinical sample was used. Samples were liquefied using an equal volume of thermo-protection buffer containing DTT. One-milliliter aliquots were heat inactivated in 2-ml screw-cap tubes at 99°C for 30 min. Cells were collected by centrifugation (6,000 × *g* for 3 min) and the supernatant discarded. Then, cell pellets were combined (if >1 available per sample) in a total volume of 1 ml PBS. Cells were again collected by centrifugation (6,000 × *g* for 3 min) and resuspended in PBS followed by another centrifugation. The final cell pellet was resuspended in 100 μl PBS, and then total DNA was extracted.

### Total DNA extraction.

The 0.08- to 0.1-g silica beads (Lysing Matrix B; MP Biomedicals, CA, USA) were added to the heat-inactivated cell suspension, which underwent two rounds of mechanical disruption using an MP Bio FastPrep-24 machine (MP Biomedicals, CA, USA) at 6.0 m/s for 40 s (5-min interval). After centrifugation at 16,000 × *g* for 10 min at room temperature, up to 100 μl of supernatant was transferred to a fresh tube (1.5-ml DNA LoBind; Eppendorf, Hamburg, Germany). DNA in the supernatant was purified using an equal volume of Agencourt AMPure XP beads (Beckman Coulter, CA, USA) incubated with mixing at room temperature for 10 min. Beads with DNA bound were magnetically separated, and the clear supernatant was removed. Beads were washed using 200 μl freshly prepared 70% ethanol and removed after a 20-s incubation. This step was repeated, and then beads were air dried for <1 min. DNA was eluted in 50 μl 1× Tris-EDTA (TE) buffer (pH 8) (Sigma-Aldrich, MO, USA) at 35°C for 10 min. DNA concentration was measured by Qubit fluorometer (Invitrogen, CA, USA), and the DNA integrity number (DIN) and fragment size were measured by TapeStation (Agilent, CA, USA).

### ONT library preparation and sequencing.

Undigested DNA (up to 90 ng) was prepared for ONT sequencing using the ligation sequencing kit (SQK-LSK109). When samples were run multiplexed (more than one per flow cell), the native barcoding expansion kit (EXP-NBD104) was used. The manufacturer’s protocols “genomic DNA by ligation” and “native barcoding genomic DNA” were followed with minor amendments; a 0.8× volume of AMPure XP beads was used to purify the end-prep and barcode ligation reactions, incubation time with AMPure XP beads was doubled, and elution was performed at 35°C. Multiplexed sequencing libraries comprised 6 barcoded DNA samples, and all libraries were sequenced using R9.4.1 SpotON flow cells on GridIONs with the MinKNOW and Guppy software versions current at the time of sequencing.

### Bioinformatics.

Nanopore reads were basecalled using Guppy (Oxford Nanopore Technology, Oxford, UK). When one sample was sequenced per flow cell (without multiplexing), all of the reads in the sequence data were analyzed. For multiplexed runs with more than one sample per flow cell, we used Porechop v0.2.2 (https://github.com/rrwick/Porechop) to perform stringent barcode demultiplexing to minimize the number of misclassified reads. Porechop searches for the presence of the barcode sequence at both the start and end of each read. Reads were classified only if the same barcode was found at both ends; otherwise, the read was discarded. This level of stringency was achieved by setting the “require_two_barcodes” option in Porechop and setting the threshold for the barcode score at 60. Porechop was used because much of the sequencing was performed prior to the availability of deepbinner or guppy_barcoder.

To allow the correct identification of M. tuberculosis complex reads from the sequencing data, we used both taxonomical classification and mapping approaches. Firstly, reads from each sample were taxonomically classified against the RefSeq database using Centrifuge v1.0.3 (22). A read was considered as candidate M. tuberculosis complex if it was uniquely assigned to a species within the M. tuberculosis complex or assigned to more than one species within the M. tuberculosis complex. Human reads were discarded and not retained as part of our in-house CRuMPIT workflow (23). Then, reads were mapped to either BCG (GenBank accession number AM408590.1; the 16S rRNA region [1498360, 1499896] was masked) or TB (GenBank accession number NC_000962.2; the 16S rRNA region [1471846, 1473382] was masked) reference sequences using Minimap2 (24) depending on the type of the sample. Reads were retained if more than 85% of the bases were mapped, i.e., if the length of a read was 1,000 bp; >850 bp were required to be mapped to the reference sequence. Finally, M. tuberculosis complex reads were identified as those agreed upon by Centrifuge and mapping. Integrative Genomics Viewer was used to view the mapping profiles (25). The mapping coverage and depth across the whole genome and 22 genes associated with susceptibility/resistance to clinically important antimicrobials (26) were analyzed using SAMtools and Pysam (https://github.com/pysam-developers/pysam).

## RESULTS

Our initial experiments focused on identifying a buffer in which mycobacteria DNA was protected from degradation during heat inactivation. Living organisms can survive at temperatures around the boiling point of water (27), indicating that DNA can exist intact at high temperatures. The high concentrations of KCl and MgCl_2_ found in some hyperthermophiles are thought to help protect their DNA against thermodegradation. This has been reproduced *in vitro* using plasmid DNA (27, 28) and formed the basis of buffer optimization experiments.

### Optimization of DNA thermo-protection buffer composition and heating duration.

**FIG 1:**
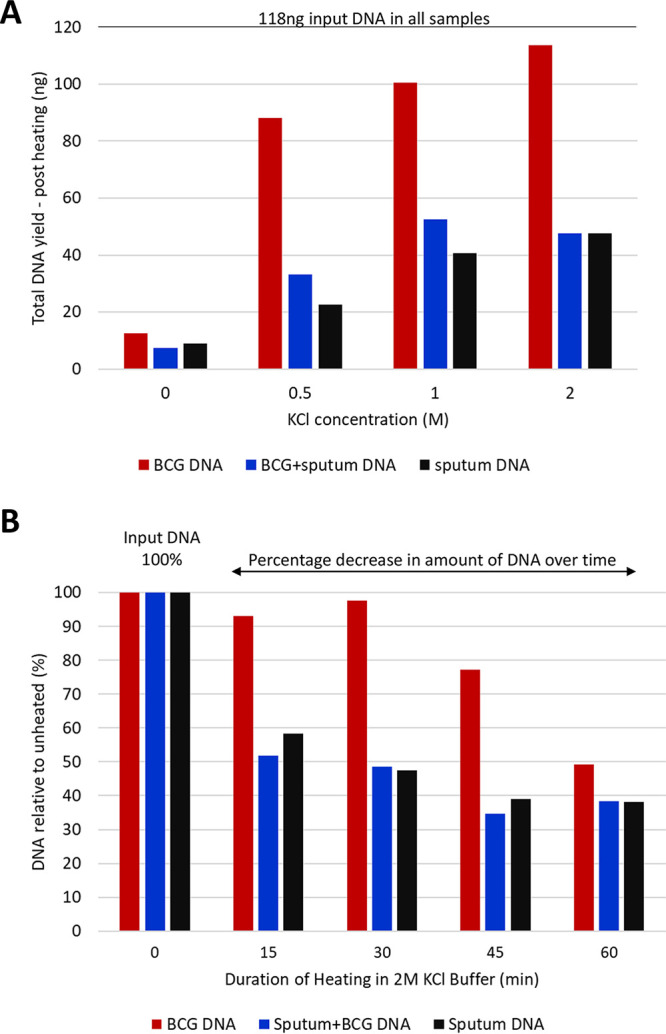
Optimization of DNA thermo-protection buffer composition and duration of heat inactivation at 99°C. (A) Extracted DNA was heated in 25 mM HEPES buffer, pH 7.5, containing 0, 0.5, 1, or 2 M KCl. Input DNA comprised 118 ng of (i) BCG DNA, (ii) BCG and sputum DNA, (iii) sputum DNA. Each DNA type was heated at 99°C, for 30 min. (B) Impact of heating duration on DNA yield. DNA remaining post heating is expressed as a percentage of the input DNA for (i) 10^5^ BCG cells, (ii) 1 ml sputum spiked with 10^5^ BCG cells, or (iii) 1 ml sputum. BCG DNA degraded more slowly than sputum DNA, indicating the potential for enrichment relative to human DNA at earlier time points.

Three different 118-ng DNA extracts were made as follows: (i) BCG DNA, (ii) BCG and sputum DNA, and (iii) sputum DNA. These were heated at 99°C for 30 min (Oxford Clinical Microbiology Laboratory health and safety requirement) in four different buffers, 25 mM HEPES, pH 7.5, plus 0, 0.5, 1, or 2 M KCl, and then DNA (ng) remaining postheating was recorded (Fig. 1A). This increased with increasing KCl (Fig. 1A). Furthermore, BCG DNA was better protected than sputum DNA; at 2 M KCl, minimal BCG DNA degradation occurred, while >50% of sputum DNA degraded (Fig. 1A), indicating potential for BCG enrichment.

Next, we determined the impact of heating duration (0, 15, 30, 45, or 60 min at 99°C) on the same three extracted DNAs in 2 M KCl thermo-protection buffer. The percentage decrease in DNA remaining after heating was plotted relative to input DNA (Fig. 1B). DNA declined over time, but BCG DNA was again more heat stable than sputum DNA. The 30-min time point was identified as optimal for BCG enrichment and meeting health and safety requirements.

### Thermo-protection of DNA in intact BCG cells.

**FIG 2:**
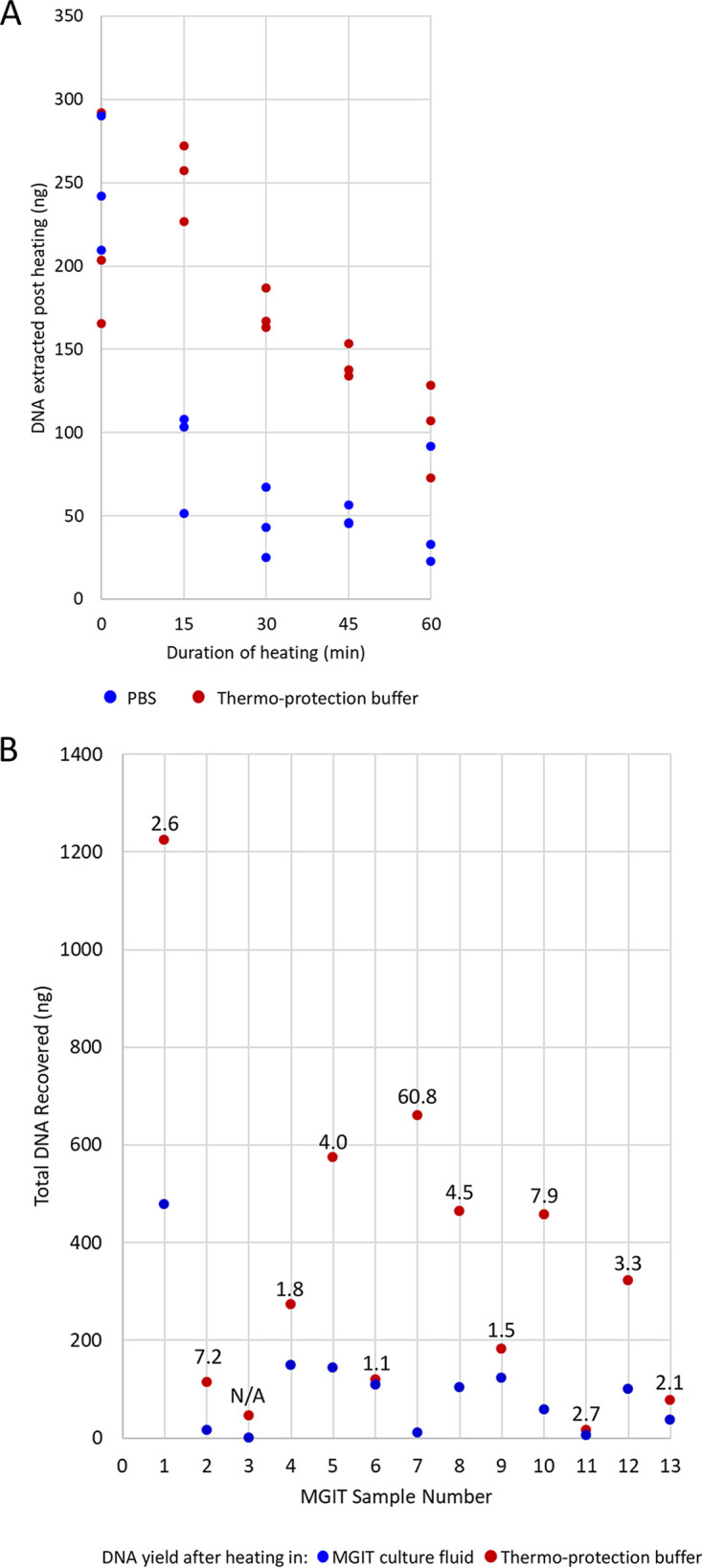
Thermo-protection of DNA in intact BCG cells. (A) Effect on DNA yield of heating intact enumerated “declumped” BCG cells in thermo-protection buffer for the times shown. A total of 10^5^ BCG cells was heated at 99°C for 0, 15, 30, 45, or 60 min in 2 M KCl and 25 mM HEPES, pH 7.5, (1× thermo-protection buffer) or PBS (control). The experiment was performed in triplicate, and DNA was extracted post heating. (B) DNA yield obtained when heating intact mycobacteria cells from positive MGIT culture in thermo-protection buffer versus heating in MGIT culture fluid. Data are shown for 13 positive MGIT cultures. The DNA yield obtained after heating for 30 min at 99°C in thermo-protection buffer compared to that after heating in MGIT fluid is plotted. Each dot indicates the total DNA recovered (ng) from 1 ml initial MGIT culture. Numbers above the dots indicate the fold improvement in DNA yield when thermo-protection buffer was used rather than MGIT culture fluid. N/A, a sample where no DNA was recovered after heating in MGIT culture fluid so no fold improvement could be calculated.

Next, we investigated whether DNA within intact mycobacteria cells could be protected by thermo-protection buffer. BCG cells (10^5^ in total) were suspended in 1 ml thermo-protection buffer and incubated at 99°C for 0, 15, 30, 45, or 60 min. Control cells were heated for the same times in phosphate-buffered saline (PBS). The experiment was performed in triplicate, and then the DNA was extracted. The DNA yield from BCG cells heated in thermo-protection buffer was markedly higher than that heated in PBS (Fig. 2A) except at time zero without heating. Here, the yield of DNA was lower than expected because the unheated cell pellet was diffuse, and cells were more easily lost than in PBS.

In a separate experiment using intact mycobacteria cells, 13 positive MGIT cultures (anonymized discards obtained from Oxford Clinical Microbiology Laboratory) were heated in 1 ml thermo-protection buffer or in culture fluid. DNA yield was improved for the cells heated in thermo-protection buffer (Fig. 2B).

### Confirmation of mycobacteria heat inactivation.

A validation experiment was performed to confirm that viable mycobacteria (M. tuberculosis H37Rv or BCG Pasteur) did not survive 30 min of heating at 99°C in thermo-protection buffer. After 8 weeks incubation at 37°C, no growth occurred in the heated samples. In contrast, room temperature controls remained viable (Table 1).

### Direct-from-sample sequencing of BCG-spiked mock clinical samples.

Four sets of mock clinical samples were made, each containing a 10-fold dilution series of enumerated BCG cells (10^5^ to 10^1^ and zero cells) in 1 ml infection-negative human sputum, liquefied in thermo-protection buffer. Four different batches of pooled sputum were used, but BCG cells were from the same enumerated batch. All four replicates (experiments A to D) underwent heat inactivation (99°C for 30 min), DNA extraction, and sequencing on the ONT platform using a single flow cell per sample. Replicates in experiments B, C, and D underwent additional microscopy (ZN staining) and GeneXpert PCR.

**FIG 3:**
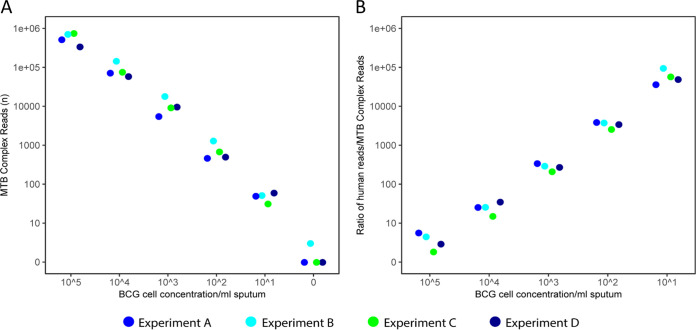
Validation of DNA thermo-protection method using mock clinical samples. Mock clinical samples containing enumerated BCG cells (0 to 10^5^) in 1 ml infection-negative human sputum liquefied in thermo-protection buffer underwent heat-inactivation at 99°C for 30 min. DNA was extracted and sequenced on an ONT MinION (1 R9.4.1 flow cell per sample). Reproducibility was assessed using four replicate experiments (experiments A to D). (A) Number of M. tuberculosis (MTB) complex reads generated per sample was linear and indicated a detection limit of 10^1^ BCG cells. (B) Ratio of human reads to M. tuberculosis complex reads.

Sequencing, microscopy, and GeneXpert PCR yielded reproducible data across the replicate experiments (Fig. 3; Table 2). The number of M. tuberculosis complex sequencing reads generated per sample was linear and indicated detection down to 10^1^ input BCG cells. At this concentration, 31, 49, 51, and 59 M. tuberculosis complex reads were detected (Fig. 3A). At 10^3^ input BCG cells, genome coverage (1×) was >90% (Table 2). The ratio of human reads to M. tuberculosis complex reads was also linear and reproducible (Fig. 3B).

**TABLE 2:** Reproducibility and detection limits of microscopy, GeneXpert, and direct-from-sample sequencing^*a*^

BCG cells	Microscopy (ZN stain)^*b*^	GeneXpert		Nanopore			
		*C_T_* value	Detection	M. tuberculosis complex reads (n)	Mean depth	Genome covered 1× (%)	Genome covered 5× (%)
Expt B							
10^5^	+++	16.4	High	701,436	261.99	98.73	98.12
10^4^	+++	16.5	High	142,918	55.0	98.30	97.75
10^3^	+	17.1	Medium	17,798	7.17	97.86	83.56
10^2^	(+)	19.3	Low	1,280	0.56	44.26	0.04
10^1^	−	23.0	Very Low	51	0.03	2.92	0
0	−		Negative	3	0	0.06	0
Expt C							
10^5^	+++	16.2	High	738,605	225.73	98.48	97.91
10^4^	+++	16.3	High	74,731	20.19	97.76	97.12
10^3^	+	17.1	Medium	9,086	2.75	91.84	17.98
10^2^	+	17.6	Medium	672	0.21	19.11	0
10^1^	−	22.7	Low	31	0.01	1.38	0
0	−		Negative	0	0	0	0
Expt D							
10^5^	+++	16.2	High	333,937	99.92	98.15	97.62
10^4^	++	16.4	High	57,824	16.28	97.66	97.13
10^3^	+	17.0	Medium	9,533	3.37	93.97	29.65
10^2^	+	18.9	Low	494	0.19	17.51	0
10^1^	−	23.0	Very Low	59	0.02	2.29	0
0	−		Negative	0	0	0	0

a Input comprised mock clinical samples. These were pooled infection-negative human sputum samples, liquefied using thermo-protection buffer and spiked using enumerated BCG cells.

b +++, Large numbers of cells/strong positive; ++, moderate numbers; +, scanty/weakly positive; −, negative.

Bioinformatics methods were optimized to ensure reads in negative controls (such as rRNA genes from nontarget bacterial species [29]) were not incorrectly assigned as BCG; prior to these improvements, close to 10,000 reads were incorrectly identified as M. tuberculosis complex in the negative control (see Fig. S1A in the supplemental material). After the improvements, the negative controls for experiments A, C, and D contained zero M. tuberculosis complex reads; however, three mycobacteria reads were present in the negative control of experiment B (Fig. 3A and Table 2; see also Fig. S1A).

GeneXpert (Cepheid) and microscopy results also followed the concentrations of the spiked BCG cells (Table 2). The detection limit of GeneXpert was 10^1^ BCG cells and that of microscopy was 10^2^ cells, with cells described as very scanty (1 or 2 per 100 fields) (Table 2).

### Direct-from-sample sequencing using multiplexing.

Sequencing more than one sample per flow cell (multiplexing) offers both time and cost efficiencies. To assess its feasibility, a short DNA “barcode” was ligated to each DNA sample, and then the 24 DNAs from replicate experiments A to D were sequenced at six samples per flow cell—one per replicate experiment. After sequencing, the barcodes were identified bioinformatically, and the data were assigned to their original sample. Unfortunately, the 10^1^ and 10^2^ BCG-spiked samples contained a similar number of M. tuberculosis reads to the negative control (see Fig. S1B); therefore, using this approach, the limit of detection declined 100-fold to 10^3^ BCG cells/ml sputum. This was a result of the barcodes of the BCG-positive samples being incorrectly (and unavoidably) identified as those of the negative control (Fig. S1B). The multiplexing approach was also compromised by a reduction in the total data available for analysis. Although we applied stringent barcode demultiplexing criteria, between 5.28 and 46.9% of total reads could not be reliably assigned to an input sample.

**FIG 4:**
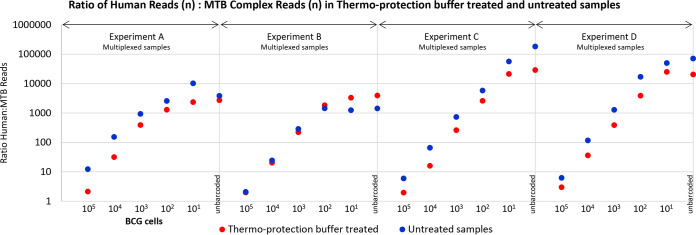
Mock clinical samples were enriched for mycobacteria DNA after heating in thermo-protection buffer. Data are shown for four replicate experiments, A to D, in which samples were barcoded and run multiplexed six per flow cell. Each experiment comprised samples made from a batch of infection-negative sputum (liquefied using thermo-protection buffer containing DTT), 1-ml aliquots of which were spiked with enumerated BCG cells at 10^5^ to 10^1^ cells and zero BCG cells (control). Sputum batch and, therefore, “background” DNA did not vary within replicates A to D, only between them. The full set of replicates was set up twice with heating (99°C, 30 min) and without heating. After sequencing, the numbers of BCG and human-derived reads were assessed and their ratio in each sample calculated. Higher ratios of human-to-M. tuberculosis (MTB) reads were obtained for samples that were not heated in thermo-protection buffer, indicating heated samples were enriched for M. tuberculosis reads relative to human reads, i.e., human DNA was depleted. The exception to this was experiment B, which yielded anomalous results because the number of reads for the unheated sample was unusually poor.

The multiplexed data for mock clinical samples in experiments A to D were also used to confirm thermo-protection buffer-associated enrichment for M. tuberculosis DNA and human DNA depletion (Fig. 4) (compared to controls prepared without heat inactivation and washing).

### Direct-from-sample sequencing of M. tuberculosis-positive clinical samples. (i) DNA preparation using thermo-protection method.

Twenty M. tuberculosis-positive clinical samples (16 sputum samples, 3 lymph node biopsies, and 1 bronchoalveolar lavage specimen) were sequenced. Samples were 1 to 14 days old, and 0.25 to 1.5 ml was available. Microscopy and GeneXpert results indicated variable M. tuberculosis loads (Table 3). The total DNA extracted ranged from 105 to 3,970 ng per sample, the DNA integrity number (DIN) ranged from 1.8 to 6.3, and the peak fragment length ranged from 1,834 to 13,949 bp (Table 3). Each sample underwent direct-from-sample sequencing using a single R9.4.1 flow cell.

**TABLE 3:** Direct sequencing of M. tuberculosis-positive clinical samples

Sample ID	Sample source^*a*^	Sample type	Vol^*c*^ (ml)	Age (days)	Parallel laboratory test		Extracted DNA			Sequence data										
					Microscopy^*d*^	GeneXpert (*C_T_*)	Total DNA (ng)	Peak fragment length (bp)	DNA integrity no.	Total reads (no.)	Human reads		M. tuberculosis complex reads		Mean depth of Coverage		Genome covered 1× (%)		Genome covered 5× (%)	
											No.	%	No.	%	Genome	R genes^*e*^	Genome	R genes^*e*^	Genome	R genes^*e*^
T15211	O	LN^*b*^	Solid	3	+++	16.2	3,590	1,834	3.3	3,755,361	2,806,761	74.74	251,256	6.69	81.02	85.59	99.60	100	99.53	100
19.0609294	B	Sputum	1	2	+	28.4	422	10,760	5.9	14,576,788	8,472,212	58.12	80,357	0.55	45.01	45.30	99.61	100	99.49	100
19.0609025	B	Sputum	1	8	NT	16.0	138	12,266	6.0	9,108,521	6,675,047	73.28	104,748	1.15	36.40	38.08	99.59	100	99.51	100
L34626	O	Sputum	0.5	1	+++	15.9	912	8,757	6.2	3,197,564	2,315,352	72.41	56,421	1.76	24.19	24.37	99.58	100	99.38	100
L32975	O	BAL	1	14	+	16.2	241	9,189	6.1	4,119,806	2,497,293	60.62	52,536	1.28	23.02	25.24	99.84	100	99.75	100
19.0608818	B	Sputum	1	12	NT	16.2	126	13,192	6.1	5,979,711	3,730,501	62.39	43,148	0.72	22.30	22.97	99.61	100	99.28	100
L87135_1	O	Sputum	1	14	+++	16.3	3,187	7,993	4.0	7,617,356	5,034,871	66.10	38,989	0.51	19.77	14.94	99.60	100	99.41	100
19.0608426	B	Sputum	1	7	+++	NT	3,970	2,403	4.4	6,635,435	6,404,796	96.52	74,672	1.13	15.74	16.62	99.40	100	98.74	100
L87135	O	Sputum	0.5	11	+++	16.3	926	6,859	3.6	5,637,884	3,569,630	63.32	26,216	0.46	14.58	14.94	99.60	100	99.10	100
L87133	O	Sputum	1	14	+++	NT	3,686	7,000	4.4	7,824,904	6,971,470	89.09	12,740	0.16	6.50	6.78	99.38	99.63	77.08	75.35
19.0609396	B	Sputum	1.5	2	+	29.3	3,206	6,900	5.6	6,719,776	6,022,913	89.63	14,491	0.22	5.89	6.23	99.05	100	71.39	77.42
L11276	O	Sputum	0.25	1	+++	16.1	1,735	8,443	5.7	5,514,521	5,397,278	97.87	9,505	0.17	4.75	3.85	98.39	99.48	53.37	32.96
L99052	O	LN	Solid	3	++	16.4	105	Could not be determined	1.8	13,507,355	13,268,163	98.23	7,124	0.05	3.36	2.90	96.03	92.19	27.27	22.47
L99521	O	Sputum	1	1	++	16.0	3,600	13,949	6.3	7,521,636	7,438,072	98.89	4,701	0.06	3.27	3.69	95.81	100	25.06	23.82
L37997	O	Sputum	0.75	3	+	16.1	228	11,879	4.0	8,014,834	7,714,434	96.25	4,650	0.06	2.89	2.83	93.14	95.02	19.39	18.37
19.0608494	B	Sputum	0.5	6	NT	16.0	2,610	1,957	3.5	11,811,075	10,915,937	92.42	8,091	0.07	2.06	2.03	86.48	83.73	7.24	6.25
L91635	O	Sputum	0.5	2	+	17.2	2,909	9,044	6.2	4,271,540	4,243,731	99.35	1,975	0.05	1.57	1.76	78.83	76.27	2.91	2.35
L96231	O	Sputum	1	8	+	NT	1,133	8,566	4.6	8,829,259	8,572,310	97.09	1,545	0.02	1.14	1.32	67.35	61.46	1.24	3.01
L11990	O	Sputum	0.25	13	+	18.5	270	6,243	5.7	4,200,973	145,749	3.47	1,898	0.05	0.86	0.73	57.53	48.04	0.34	0
W63114	O	LN	Solid	2	++	NT	265	1,990	4.7	7,408,231	7,351,842	99.24	1,825	0.02	0.55	0.71	42.97	48.74	0.05	0

a O, Microbiology Department, Oxford University Hospitals NHS Trust, Oxford, UK; B, PHE National Mycobacteria Reference Service-North and Central, Birmingham Public Health Laboratory, UK.

b LN, lymph node, solid piece of tissue, disrupted by bead beating in saline before receipt.

c Equivalent volume of initial clinical sample received.

d +++, Large numbers of cells/strong positive; ++, moderate numbers; +, scanty/weakly positive; NT, not tested.

e Resistance genes, coverage across 22 genes associated with susceptibility/resistance to clinically important antimicrobials (26).

### (ii) Sequence data.

**FIG 5:**
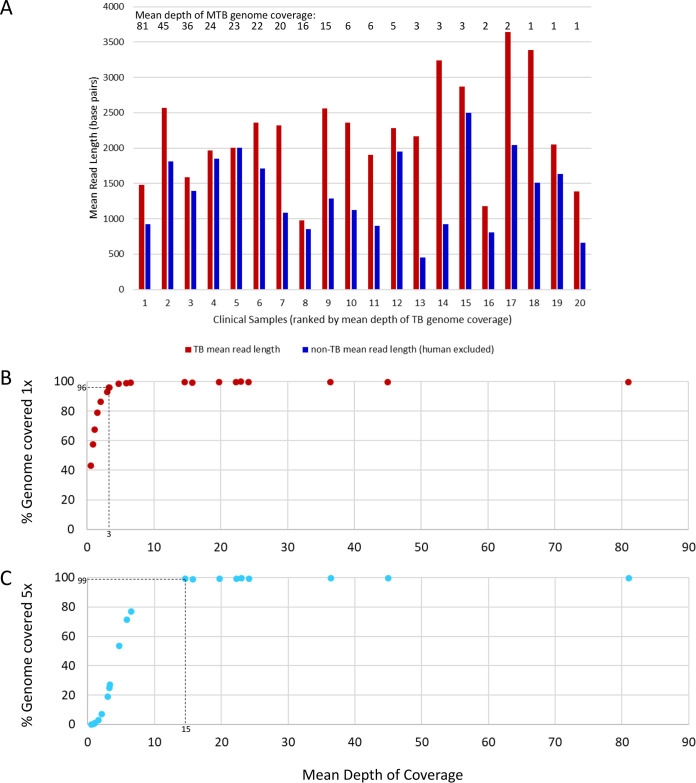
Sequence data generated direct from clinical samples—mean read lengths (M. tuberculosis [TB] versus non-M. tuberculosis nonhuman sequences) and relationship between mean depth of coverage and complete genome coverage. (A) Comparison for each of 20 clinical samples of mean read length for M. tuberculosis and non-M. tuberculosis sequences (human sequences excluded prior to analysis). Clinical samples were ranked according to mean depth of coverage, indicated by numbers above the bars. (B) Relationship between mean depth of coverage and percentage of the M. tuberculosis genome covered once. A mean depth of coverage of three is required to achieve >96% 1-fold TB genome coverage, achieved in 15/20 clinical samples. (C) Relationship between mean depth of coverage and percentage of the M. tuberculosis genome covered five times. A mean depth of coverage of 15 is required to achieve >99% 5-fold genome coverage, achieved in 9/20 clinical samples.

The total number of reads obtained per flow cell ranged from 3,197,564 to 14,576,788 (Table 3). Human reads were discarded prior to detailed analysis (ethics requirement). Among the nonhuman reads, mean M. tuberculosis read length was up to 4.77 times longer than non-M. tuberculosis (Fig. 5A). M. tuberculosis reads were detected in all 20 clinical samples (Table 3), with the mean depth of genome coverage ranging from 0.55 to 81.02. An initial sample volume of ≥1 ml and lower percentage of human reads was apparently associated with higher depth of coverage, although the numbers were too small for statistical analysis (Table 3).

Plotting the mean depth of coverage against the percentage of M. tuberculosis genome covered once or five times revealed that ≥96% of the genome was covered once when a mean depth of approximately three was reached and ≥99% was covered five times after a mean depth of 15 (Fig. 5B and C). The depth of coverage across 22 key genes used to predict susceptibility to clinically important antimicrobials (26) closely followed the mean genome coverage (Table 3) (except one bioinformatically masked rRNA gene), indicating the absence of bias and confirming potential for antimicrobial resistance prediction.

## DISCUSSION

DNA degrades rapidly at high temperatures (30); therefore, heat-inactivated M. tuberculosis clinical samples typically yield poor quality material for sequencing. Here, we describe a simple, low-cost method that overcomes this important technical issue. Sputum liquefaction and heat-inactivation were accomplished following addition of an equal volume of thermo-protection buffer (4 M KCl, 0.05 M HEPES buffer, pH 7.5, and 0.1% DTT), which inhibited DNA degradation during heat inactivation, with coincidental enrichment for mycobacteria DNA (Fig. 1, 2, and 4). Buffer addition was the only handling step involving infectious material, minimizing risk to staff in settings where containment laboratories are not available. The choice of the ONT sequencing platform reflected our aim to transfer the protocol to high-burden settings in diverse locations, lacking facilities such as air-conditioned laboratories, and where the ONT platform performs well (11–14). The characteristic ONT long read lengths (31, 32) require high-quality, ideally nonfragmented input DNA. Hence, the ONT platform provided a robust test of extracted DNA quality, indicating that the method should be transferable to other sequencing platforms such as Illumina.

The thermo-protection buffer was designed to emulate intracellular conditions of hyperthermophiles (27). At high temperatures, intracellular salts such as KCl and MgC1_2_ are thought to protect the DNA’s N-glycosidic bonds against depurination and cleavage by hydrolysis of the adjacent phosphodiester bond (15, 33, 34). We chose K^+^ over Mg^2+^ because high K^+^ concentrations protect against cleavage at apurinic sites, while high Mg^2+^ concentrations stimulate this (34). Furthermore, plasmid DNA appeared better protected in KCl (Fig. 1 in reference 34). The choice of KCl concentration (2 M) was informed by our own data (Fig. 1) and published data (27, 28). The mechanism whereby DNA in intact mycobacteria cells was protected during heating in thermo-protection buffer (Fig. 2) is unclear but suggests that the cell is or becomes permeable to K^+^ during heating. Our data (Fig. 2B) indicate that thermo-protection buffer can also improve the DNA yield obtainable from positive MGIT cultures, as currently used routinely by Public Health England (35, 36), potentially reducing sequencing failures on the Illumina platform due to low DNA yield.

Oxford clinical microbiology laboratory inactivates mycobacteria-positive samples at 99°C for 30 min because less stringent conditions (e.g., 20 min at 80°C) show variable efficacy (37–43). We confirmed that mycobacteria heated in thermo-protection buffer at 99°C for 30 min were not viable (Table 1). Sequencing direct from sample is enhanced when input DNA is enriched for sequences of interest (17–19, 44). Depletion of up to 99.99% human DNA from non-TB respiratory tract samples has been achieved using saponin, osmotic shock, and high-salt nuclease treatments (45). However, no heat-inactivation was performed, and a specialist nuclease (salt-active nuclease; ArcticZymes, Tromsø, Norway) was required. Interestingly, we observed that nontarget DNA degraded more rapidly than mycobacteria DNA during heating in thermo-protection buffer for 30 min at 99°C, providing fortuitous enrichment (Fig. 1, 2, and 4). Consistent with this, mean read length obtained for M. tuberculosis was longer than non-M. tuberculosis reads (Fig. 5A). Sufficient nontarget DNA remained in our samples to provide a useful “carrier.” This was particularly important in low-titer M. tuberculosis samples; our 10^1^ BCG limit of detection in mock clinical samples (Fig. 3A) would not have been achieved without this carrier DNA.

Three features of mycobacteria DNA may have contributed to its enrichment on heating (Fig. 1 and 4). First is a higher GC content (M. tuberculosis 65.6% GC versus <50% GC for ∼92% of human DNA) (46, 47). Second, intact M. tuberculosis chromosomes are covalently closed circles—resistant to thermo-denaturation because the two single strands remain intertwined during heating (48). Third, the M. tuberculosis chromosome is negatively supercoiled (underwound—a feature potentially connected to its slow growth rate [49]) but less so than some bacterial species, including Escherichia coli (50). Lesser negative supercoiling reduces base exposure (51), which may reduce susceptibility to thermodegradation relative to human DNA.

We obtained optimal results when using a single R9.4.1 flow cell per sample (Fig. 3; Table 3). This approach would be prohibitively expensive if used routinely since a flow cell costs £380 to £720 depending on order size (1 to 300). Unfortunately, multiplexing six samples per flow cell did not provide a solution since the inefficiency of barcode ligation and incorrect bar code identification postsequencing reduced the limit of detection 100-fold (see Fig. S1 in the supplemental material). Run-time flexibility is also incompatible with multiplexing due to variations in sample M. tuberculosis titers. Solutions may be to wash, regenerate, and reuse flow cells after each use (flow cell wash kit; Oxford Nanopore Technologies) or to adopt single-use ONT Flongles at a cost of £72.50 each (as of 30 January 2020). Unfortunately, the latter currently offers only 60 to 70 active sequencing pores in our hands compared to 1,200 to 1,500 pores per R9.4.1 flow cell.

The thermo-protection method was applied successfully to clinical samples (*n* = 20). Higher mean depth of genome coverage appeared to reflect initial sample volume (≥1 ml being ideal) and a lower human DNA content but did not necessarily correlate with microscopy or GeneXpert threshold cycle (*C_T_*) values (Table 3). This may reflect the known variation in copy numbers of GeneXpert targeted insertion sequences (IS*6110* and IS*1081*) between BCG (used in mock samples where limit of detection, microscopy, and GeneXpert data all correlated) (Table 2) and M. tuberculosis (52, 53). Also, microscopy was performed using auramine staining for clinical samples and ZN staining for mock clinical samples, and the former was performed by multiple different staff members.

The accuracy of DNA consensus sequences obtained using the Nanopore platform is 99.9% when nanopolish is used (https://github.com/rrwick/Basecalling-comparison#references), indicating potential for antimicrobial susceptibility prediction. Further work is required to examine this aspect in detail, particularly for rRNA genes, which were masked to improve the accuracy of M. tuberculosis detection but are implicated in resistance (such as the 16s rRNA *rrs* gene; aminoglycoside resistance). A further potential advantage of direct-from-sample sequencing is the detection of more genetic diversity than sequencing from culture (using SureSelect and Illumina) (54). Increasing the numbers of target reads from low-titer samples will require innovations, such as mycobacteria cell fractionation or concentration, followed by unbiased DNA amplification. Such approaches could be simplified for application in resource-poor settings if they were adapted to a cartridge-based system.

In summary, a simple, low-cost method was developed to prepare M. tuberculosis DNA for sequencing direct from clinical samples. Neither commercial kits nor time-consuming culture were required, but the key health and safety requirement heat-inactivation was retained and exploited to achieve target sequence enrichment. Available data suggest that the method can yield complete M. tuberculosis genome sequences direct from clinical samples without amplification, achieving up to 81-fold mean depth of coverage (Table 3). The protocol is currently undergoing testing by collaborators in India and Madagascar, with early data indicating reproducibility.

## Supplementary Material

Supplemental file 1
