# Supplementary material for: DNA Thermo-Protection Facilitates Whole-Genome Sequencing of Mycobacteria Direct from Clinical Samples
Source: J Clin Microbiol. 2020 Sep 22;58(10):e00670-20. doi: 10.1128/JCM.00670-20 (PMC7512152; doi:10.1128/JCM.00670-20)
Supplement: Supplemental file 1 [file JCM.00670-20-s0001.pdf]

Fig. S1

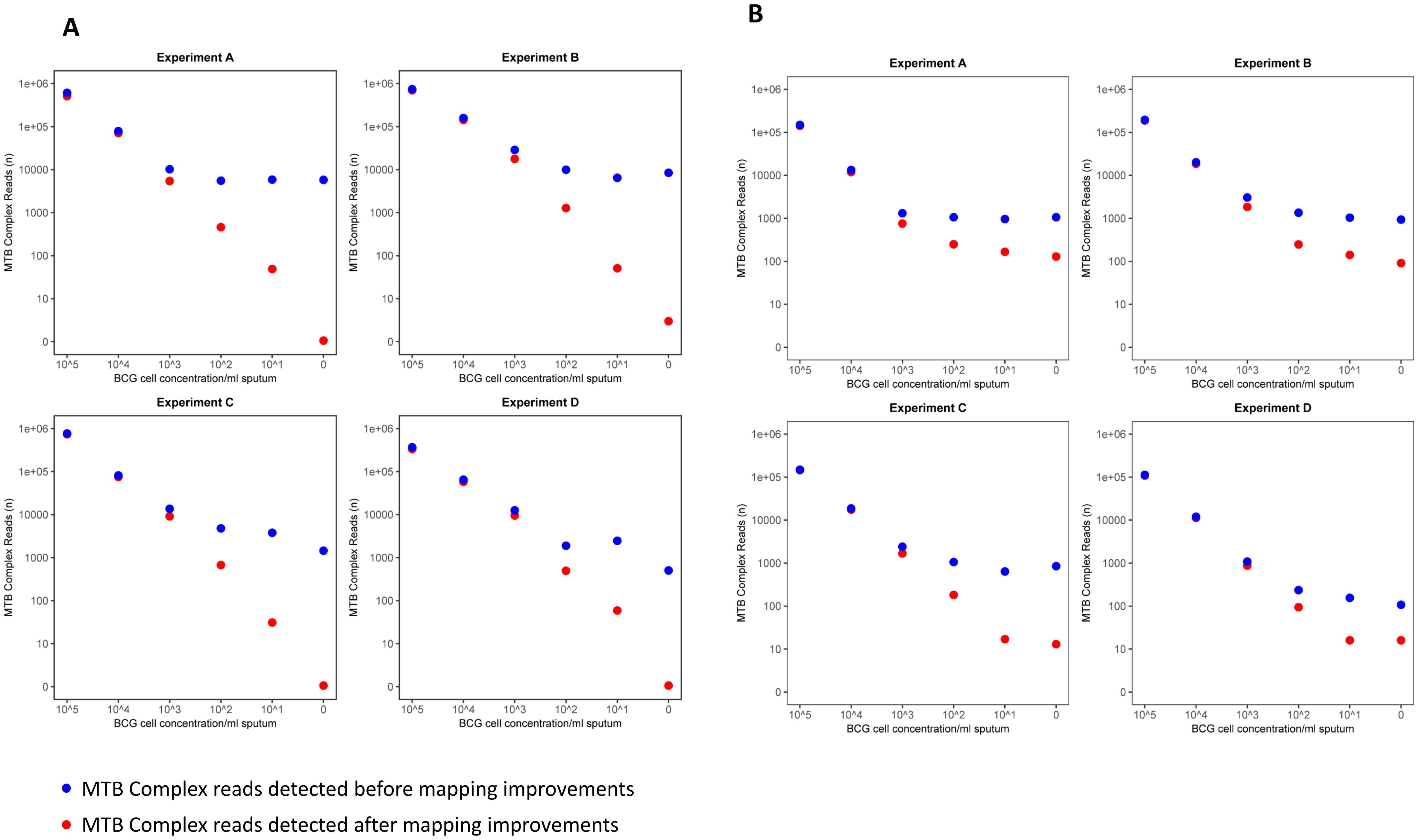

Fig. S1

Improvements to bioinformatic analysis of sequence data generated direct-from-sample.

(A) Analysis of results generated from mock clinical samples, each sequenced on a single flow cell. Samples comprised BCG spiked infection negative sputum containing 10^5 to 10^1 and zero BCG cells. Data are shown before (blue dots) and after (red dots) mapping improvements. Prior to mapping improvements, close to 10,000 reads per sample were incorrectly identified as MTB complex. After the improvements, three contaminant reads were detected in the negative control (zero BGC cells) of experiment B only. These were 612, 788, and 1305 bases long and mapped independently and at high quality to the BCG reference genome, at positions 2798622-2799216, 282379-283169 and 2701067-2702371, the identity of these reads was further confirmed by BLASTn.

(B) Analysis of results generated from mock clinical samples, ‘barcoded’ and sequenced multiplexed six per flow cell. The results are shown before (blue dots) and after (red dots) mapping improvements. Even after mapping improvements, many reads persisted in the low BCG titre samples and negative controls. So, when running multiplexed samples, the limit of detection was compromised at 10^3. The reason for this was the barcodes of DNA fragments belonging to BCG positive samples were incorrectly (and unavoidably) identified as the barcode of the negative control. The multiplexing approach was also compromised by a reduction in the total data available for analysis, since a relatively high proportion of the total reads were unbarcoded.
